# Anthropomorphic breast model repository for research and development of microwave breast imaging technologies

**DOI:** 10.1038/sdata.2018.257

**Published:** 2018-11-20

**Authors:** Muhammad Omer, Elise Fear

**Affiliations:** 1 Department of Electrical and Computer Engineering, Schulich School of Engineering, University of Calgary, Calgary, AB, Canada

**Keywords:** Biomedical engineering, Electrical and electronic engineering

## Abstract

A repository of anthropomorphic numerical breast models is made available for the scientific community to support research and development of microwave imaging technologies for diagnostic and therapeutic applications. These models are constructed from magnetic resonance imaging (MRI) scans acquired at our university hospital. Our 3D breast modelling method is used to translate the MRI scans into 3D models representing the geometry and microwave-frequency properties of tissues in the breast. The reconstructed models demonstrate anatomical realism, reconfigurable complexity, and flexibility to adapt to simulations of various microwave imaging techniques and prototype systems. With these models, realistic and rigorous test scenarios can be defined in simulations to support feasibility analysis, performance verification and design improvements of developing microwave imaging techniques, prior to testing on experimental systems. A repository of breast models is created which includes breasts of varying classification – fatty, scattered, heterogeneous, and dense. In addition, the models include brief documentation to facilitate researchers in selecting a model by matching its features with their requirements.

## Background & Summary

The research and development of microwave breast imaging and therapeutic technologies has greatly benefitted from MRI-derived anatomically realistic numerical breast models^1–8^. These models offer several advantages over experimental breast phantoms, including capturing a high degree of realism in terms of shape, size, internal structure, and heterogeneity of the tissue properties. In addition, these models provide the flexibility of embedding tumours of varying type, size, and morphology at different locations. This allows the generation of a large number of test cases to conduct rigorous testing, feasibility analysis, performance verification, and design improvements for microwave breast imaging techniques.

A 3D numerical breast model is similar to a three-dimensional data matrix, where each element of the matrix, known as a voxel, signifies a certain tissue at a specific location inside a human breast. These models can be conveniently constructed from breast MRI scans, as these images provide information on the spatial distribution of tissues in the breast. Creating microwave-frequency models from MRI images also involves mapping pixel intensities in the MRI scans to dielectric properties for the microwave models.

Several methods have been proposed to construct numerical breast models from the MRI scans, employing various image processing techniques^9–12^. A notable method is presented in^9^, with an associated breast model repository^13^. These models have been widely used to investigate microwave imaging techniques. Recently, we introduced methods to generate models using a pipeline that takes advantage of 3D processing methods to reduce artefacts and promote anatomical connectivity. The modelling approach also enables the construction of adjustable models with reconfigurable model complexity or shape.

In this work, we present a new repository of high quality breast models derived for the first time from 3.0T MRI scans. The aim of this repository is to provide the research community with an expanded set of models. We apply our 3D breast modelling method^14^ to construct models demonstrating anatomically realistic shape and structural connectivity. Our breast model repository has several features which are expected to enhance its applicability and convenience. First, the model elements are created as modules (clusters), enabling reconfigurable complexity and adaptability in terms of sizes and shapes. Second, the repository contains models of both left and right breasts of each volunteer, which is favourable for developing differential imaging algorithms. Third, the models are available in multiple file formats, compatible with a wide range of computational software packages. Furthermore, each model has associated pre-computed tissue property files, which are readily importable in simulation software. All of these model attributes enhance convenience and applicability throughout the developmental stages of imaging technologies, ranging from initial idea verification to advanced design, analysis and rigorous testing. For example, these models have supported recent development of both microwave radar and microwave tomography imaging techniques^2,15–17^.

## Methods

### Acquisition of MRI Scans

To construct a repository of anthropomorphic numerical breast models, high resolution MRI scans were selected. These scans were acquired at our university hospital (University of Calgary, Foothills Campus) as part of a patient study involving microwave imaging prototype systems. This study was approved by University of Calgary Conjoint Health Research Ethics Board (REB15-1118 and E22121) and informed consent was obtained from all subjects. The MRI scans served as the ground truth to assist in the interpretation of the microwave imaging results. Moreover, these scans were used to construct anatomically realistic numerical breast models, which are important tools for research and development of novel medical imaging technologies.

The 3.0T MRI scans were acquired using General Electric’s (GE) VIBRANT Flex technique and a dedicated breast coil. This involves use of a Gradient Recalled Echo (GRE) pulse sequence with a Repetition Time (TR) of 4.654 ms and dual Echo Times (TE) of 2.66 ms and 1.392 ms, to form in-phase and out-of-phase images, respectively, and a flip angle of 10 degrees. This scan sequence enables fat-free imaging to reveal underlying breast anatomy. Each scan contained 240 images or slices in Digital Imaging and Communications in Medicine (DICOM) format, where the dimension of each slice is 256 × 256 pixels. These 2D slices are merged together to form a 3D volume, where the spatial extent of each voxel (element) is 0.9375 × 0.9375 × 1.1 mm^3^.

A full spectrum of breast classification is covered including fatty, scattered density, heterogeneously dense, and dense breast models. The 3.0 T MRI scans available for the construction of model repository included healthy volunteers only. However, a single 1.5 T MRI scan with a tumour was available^18^. This scan was also processed to construct a model of a diseased breast (BM_Fatty_001/Left, Data Citation 1). In addition, the breast tumour from this scan was segmented and made available as a separate model element for the researchers to include with healthy breast models and simulate a diseased breast. Table 1 summarizes the study input, processing technique, and data output for the model repository.

### Modelling Method

**Figure 1:**
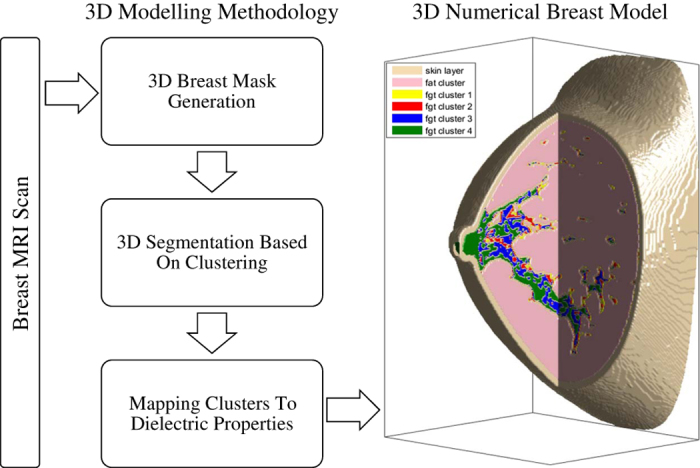
Flowchart of the steps involved in the construction of anthropomorphic numerical breast models. The breast MRI scan is the study input, which is fed to the 3D breast modelling method for processing. The output data is 3D numerical breast model comprised of tissue clusters, including skin, fat and single or multiple fibroglandular clusters.

The 3D breast modelling method employs various volume processing techniques to transform the MRI scans to 3D numerical models. This method has previously been described in detail^14^. In the first stage, a 3D breast mask is generated to identify the breast region from the background. This is achieved by traversing the scan in three orthogonal planes (transverse, sagittal and coronal) while sensing the breast surface using a threshold value computed from the background voxels. The breast surface is constructed by taking the intersection of the three surfaces identified across each orthogonal plane. Once the breast region is identified, a clustering method is applied in stage two to segment the breast tissues into skin, fat and fibroglandular clusters. The segmented tissues can be further sub-clustered to add reconfigurability to the reconstructed models, where the increasing number of sub-clusters implies increasing level of model complexity (tissue heterogeneity). Since the fibroglandular tissues of the breast have significant heterogeneity in dielectric properties, we create multiple models with varying number of fibroglandular sub-clusters. In contrast, the fat tissues of the breast are fairly homogeneous in terms of the dielectric properties and hence they are represented by a single fat cluster. The various stages of the breast modelling method are shown in Fig. 1.

The outcome of the first two stages of our modelling methodology is the breast region segmented into a set of 3D model elements (clusters), including skin, fat and fibroglandular clusters. Each element of this set is mutually exclusive of the other elements, providing ability to process each breast tissue independently. The processing may include sub-clustering of the model elements to add complexity to the model or applying tissue specific deformation to emulate physical effects of experimental systems. The processed clusters can then be assembled to construct a model with desired features.

To complete the model construction for microwave imaging applications, the clusters are assigned dielectric tissue properties using a linear mapping strategy^9^. This involves defining voxel intensity ranges for different tissues based on the statistics of model intensities and assigning dielectric property curves to each range using the values published in the literature^19^. The average voxel intensities of the clusters are computed and compared with the intensity ranges to determine the weighting factor with which property curves are combined to yield their dielectric properties.

It should be noted that the clustering approach will not allow the model to achieve the highest degree of heterogeneity. However, allowing the user control on the model complexity may benefit the development process of new imaging technologies. For instance, it provides the user an ability to design a set of experiments with progressively increasing level of breast heterogeneity to verify the robustness of the imaging algorithms.

In addition, such models can also be used to analyse the impact of model complexity on microwave imaging signals to quantify their sensitivity in resolving fine structural details of the breast. A preliminary study^15^ conducted with these models showed a difference between scattered microwave responses acquired from single (homogeneous) and multiple (heterogenous) clusters models. However, a high degree of similarity in scattered responses was observed between models with 8 and 16 fibroglandular clusters. This understanding can be used to design simple yet realistic numerical models with heterogeneity levels which microwave signals are realistically expected to resolve. In addition, the knowledge of this can also help us later in developing experimental phantoms with limited yet realistic heterogeneity.

Another advantage of modelling breast tissues as clusters is their suitability for commercially available full wave electromagnetic softwares. The model clusters can conveniently be imported as 3D objects and assigned dielectric properties. These objects can also be easily transformed according to the simulation requirements.

### Code Availability

The breast model repository includes example scripts to demonstrate its usage. A detailed description of these scripts, coding languages and functionalities can be found in the Usage Notes section.

## Data Records

**Figure 2:**
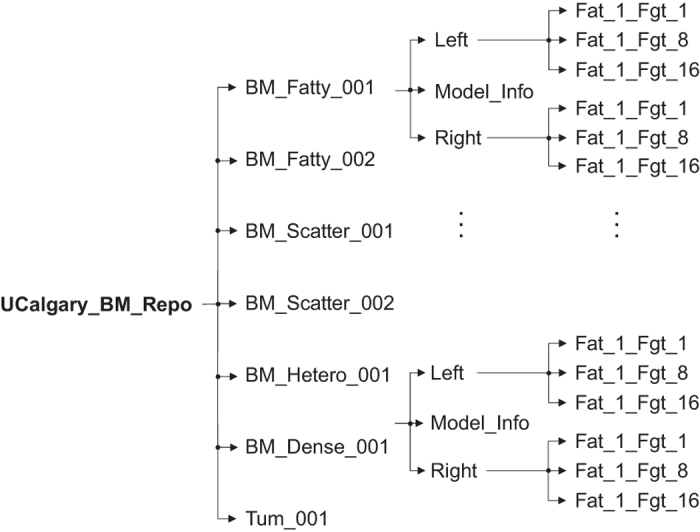
Directory structure of the breast model’s repository, depicting folders and sub-folders of models of each breast class. The folder names follow the convention of BM_Class_ID. Each folder contains two sub-folders corresponding to breast side. The breast side folders further contain three sub-folders each, containing models of varying number of fibroglandular clusters. The ‘Model_Info.pdf’ file contains useful information about the model, such as breast classification, density, dielectric properties and visualization.

The model repository contains several anthropomorphic numerical breast models covering a full spectrum of breast classifications (Data Citation 1). This model repository extends the range and variety of breast models reported in our earlier work on breast modelling method^14^, including four additional models of scattered and heterogenous classification. The directory structure of the full model repository, named University of Calgary Breast Model Repository (UCalgary_BM_Repo, Data Citation 1), is shown in Fig. 2. The repository contains a root folder (\UCalgary_BM_Repo) and its sub-folders (called model folders) correspond to the models of varying breast classification. The naming of the model folders follows the convention of BM_Class_ID, where BM is the abbreviation for breast model, Class represents breast classification and ID is a number assigned to each model of this class. For instance, BM_Fatty_001 (Data Citation 1) folder corresponds to the first breast model of ‘Fatty’ classification. Each model folder contains left and right sub-folders, called model sides folder, corresponding to each side of the breast (Left/Right, Data Citation 1). These folders further contain three sub-folders (called model elements folder), containing model elements with varying numbers of fibroglandular clusters. The root folder also contains a folder (ExampleScripts, Data Citation 1) that includes sample scripts to demonstrate the usage of the model repository in constructing a numerical breast phantom. More information about these scripts is included in the Usage Notes section.

**Figure 3:**
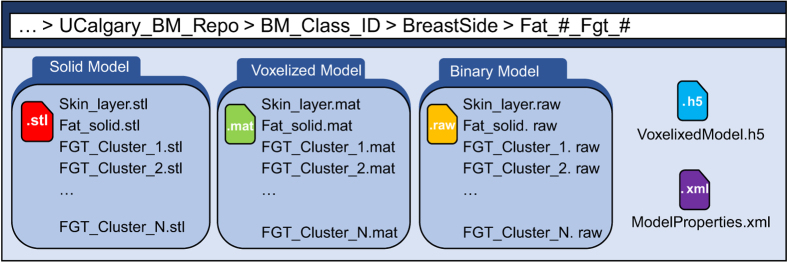
Sub-folders and contents of each model element folder, containing model in three data formats, including the STL, MAT and RAW formats. The voxelized model is also available in HDF5 format and the corresponding properties are stored in the XML format model properties file

The model clusters folder (Fat_#_Fgt_#, Data Citation 1) contains three sub-folders, namely, ‘Solid Model’, ‘Voxelized Model’ and ‘BinaryModel’ as shown in Fig. 3. The first folder (SolidModel, Data Citation 1) contains model files in stereolithographic (STL) format, which is compatible with commercial computational electromagnetic simulations software. The next folder (VoxelizedModel, Data Citation 1) contains model files as voxelized data in Matlab data format (MAT). This format is convenient for researchers using Matlab-based custom built 2D or 3D electromagnetic solvers. A complete model can be easily imported into a simulation space as a 3D data matrix for full 3D simulations while 2D slices (matrices) can also be extracted for simpler and faster 2D simulations. The third folder (BinaryModel, Data Citation 1) contains the model files in raw binary format (RAW), which is another representation of the voxelized model as binarized data and is supported by many computational electromagnetic simulations software packages. This model format provides advantages of efficient storage, transfer and language independent access.

**Figure 4:**
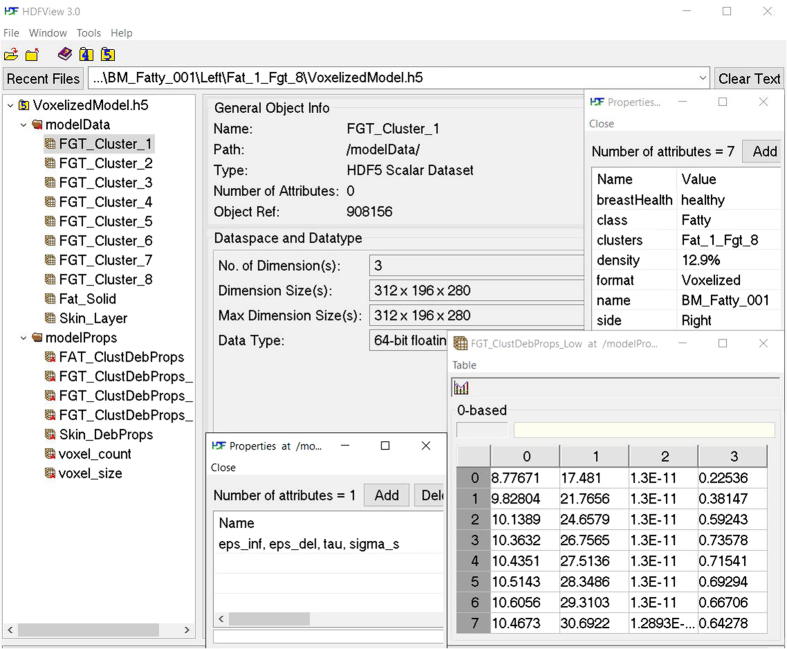
Directory structure of the voxelized breast model in HDF5 file format. This format enables convenient structuring of the model elements as groups (e.g. modelData), elements (e.g. FGT_Cluster_1), and attributes (e.g. breastHealth). In addition, it also enables storage of 3D model elements as 3D data matrices.

Another model format selected for model representation is called Hierarchical Data Format 5 (HDF5)^20^. This file (VoxelizedModel.h5, Data Citation 1) represents the voxel based model stored in this data format. The application programming interface (API) provided by HDF5 developers is used to construct a single model file containing model data and properties as groups and elements while related model features such as breast health, class, density, name, and number of tissue clusters etc. are included as attributes. The HDF5 format was selected due to its portability and suitability to store large and complex data. In addition, it is a language independent format supported by many software packages that allows for greater accessibility and reuse value of the data. In addition, the developers provide a viewer software (HDFView) for quick visualization, browsing and editing of data. Fig. 4 shows an example of a breast model in HDF5 file format opened in an HDFView. A summary of available model data, property formats, types and compatibility with simulation software is included in Table 2.

The availability of model elements as 3D clusters enables simulation of breast deformation to emulate real imaging scenarios, such as breast compression during breast mammogram acquisition. The voxelized model can allow only basic model deformations, achievable by scaling voxel sizes in desired dimensions. However, the STL based model can be used to model complex deformations. This format can be imported as a finite element model and converted into a biomechanical model by assigning tissue specific elastic properties. A number of finite element method based computational techniques reported in the literature^21–24^ can be used to estimate breast deformation due to an external stimulus.

**Figure 5:**
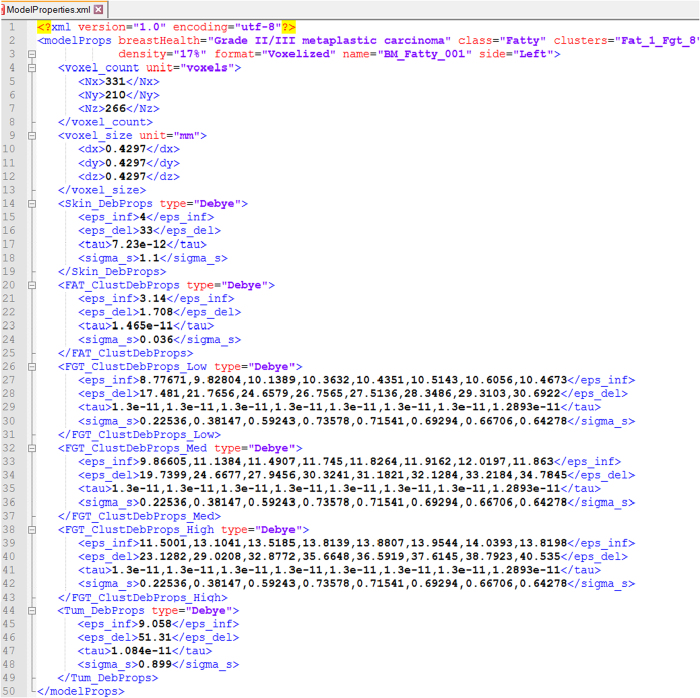
Contents of model property file available in XML format. The format includes a root node (modelProps), attributes (e.g. breastHealth) and element nodes (e.g. FGT_Clust_DebProps). The element nodes further have child nodes (e.g. eps_inf) which are assigned corresponding property values.

The last file of the model clusters folder (ModelProperties.xml, Data Citation 1) contains model properties associated with the model elements in Extensible Markup Language (XML) format. The XML file format allows the creation of custom markups (or tags) to define a data structure and include data as elements or attributes. The features provided by this data format are used to integrate all model properties in a single file. The properties such as voxel count, voxel size and electromagnetic properties of various breast tissues are included as model elements while the model features such as breast health, class, density, name, and number of tissue clusters are included as attributes. The complex dielectric permittivities of the model elements are defined using a single pole Debye model. It is comprised of four entries, which are the high frequency permittivity *ε*_∞_, Δ*ε=ε*_*s*_−*ε*_∞_ (*ε*_*s*_ is the static or low frequency permittivity), relaxation time τ and static conductivity σ_s_. The model property files contain tags for each breast tissue (e.g. <Skin_Layer > ), where each tag further contains four Debye parameter element tags (e.g. <eps_inf > ) and their corresponding values. An example of a model property file showing all property tags is shown in Fig. 5.

A key challenge with microwave breast imaging is the detection of anomalies which are typically located in the fibroglandular tissue. This detection is sensitive to the contrast between the fibroglandular tissues and tumour. The repository includes a range of contrasts for the model, which are obtained by scaling the average properties of the fibroglandular clusters. Since the fibroglandular tissues are the most heterogenous tissues of the breast, we provide three property levels, namely FGT_ClustDepProps_High, FGT_ClustDepProps_Med and FGT_ClustDepProps_Low. The high dielectric properties are obtained by linearly mapping the mean pixel intensities of the fibroglandular clusters to the dielectric property curves presented in the literature^9^. In contrast, the medium and low property values are obtained by first scaling the dielectric property curves^9^ by factors of 0.85 and 0.75, respectively, followed by adopting the same linear mapping methodology. These property files represent three variations of dielectric property distributions for the fibroglandular tissues, which can be used to build simulation models of the same geometry but different heterogeneity, loss and dielectric contrast (ratio of dielectric properties of tumour and fibroglandular tissues).

An additional file (Model_Info.pdf, Data Citation 1) is included with each model folder, which contains a brief and important description of the various model features. The document is divided into four sections. The first section contains general model information, such as volunteer ID, breast classification, intensity histogram and a list of available models. This is followed by the model elements section, which includes information about the grid size, paths of model clusters, inclusions and available model formats. The third section includes extensive plots of the frequency dependent dielectric properties of the model elements for various model variations, including different numbers of fibroglandular clusters and property files. The last section presents visualization of the model elements, showing dielectric property maps for all model variations and a 3D model visualization. This comprehensive information is included to help the users in selecting the models which best serve their simulation requirements.

To facilitate inclusion of realistic tumour models, another folder (Tum_001, Data Citation 1) is included with the repository, containing a numerical model of a tumour in solid, voxelized and binary formats. Since the 3.0T MRI scans acquired during this study included only healthy volunteers, a 1.5T MRI scan^18^ containing malignant tumour of type Grade II/III metaplastic carcinoma and 10 mm diameter in size, is used to segment the tumour volume through manual cropping and thresholding techniques. The segmented tumour region is assigned sample dielectric properties of a malignant tumour^19^. All this information is also included in the model properties files for user’s reference.

## Technical Validation

The purpose of constructing anthropomorphic numerical breast models is to enable design, development and testing of new breast imaging techniques. The anatomical structural details are extracted from the high-resolution MRI scans, while the dielectric properties of the tissues are computed using a previously proposed approach of linearly mapping voxel intensities to tissue properties. However, the reconstructed breast models are approximate representation of actual breast tissues, in both anatomy and tissue properties. Validation of these models may require direct measurements of the volunteer’s tissue properties during surgery, however these measurements are not performed for our healthy volunteers or patients. For both measurements performed during surgery and on excised tissue samples, mapping the location of the measured tissue to a particular location in the MRI scan or model is challenging. Despite challenges with directly validating the models, segmenting images and mapping dielectric properties to pixel intensities is the accepted approach to creating models for EM simulations^9,25^. The numerical models reported in this paper provide researchers with flexible tools to create simulations of varying complexity for robust testing of breast imaging algorithms.

To ensure the high quality of the reconstructed breast models, the following measures are taken. First, the MRI scans are acquired using a 3.0T scanner and a sequence revealing high resolution anatomical details. Secondly, the scans are processed with our novel 3D breast modelling method^14^, which extracts realistic breast shapes and maintains inter-slice connectivity of the fibroglandular tissues. Lastly, the range of dielectric properties assigned to the model elements is consistent with the properties currently being used in the existing models^9,19^ employed for microwave imaging research.

The anthropomorphic numerical breast models are constructed using MRI scans providing high resolution images, high contrast between breast tissues and reduced imaging artefacts. For this purpose, we use the Discovery^TM^ MR750, a 3.0T system (GE Healthcare, USA), which is applied in advanced clinical and academic studies due to its high precision and performance. It uses a time-efficient dual-echo acquisition scan sequence to simultaneously reconstruct in-phase and out-of-phase scans, resulting in an artefact-free fat suppression. Moreover, an Auto-Calibrating Reconstruction (ARC) parallel imaging technique enables faster data acquisition and reduced times, leading to reduction in motion related artefacts. The result of all these scan attributes is a clear depiction of underlying breast anatomy.

The resulting MRI scans, however, are affected by ringing artefacts (Gibbs Phenomenon). These artefacts are reduced with our 3D breast modelling method, where a principle component analysis (PCA) method is applied to extract important structural information of the scan while discarding weak components, such as ringing artefacts and background noise^14^. The denoised data is further processed by a number of 3D processing techniques to yield breast models of realistic shapes and structure.

Since the study did not involve annotation of the volunteers MRI scans, the accuracy of the anatomical structure of the reconstructed models is verified by comparing the breast density computed from the segmented breast tissues with the radiologist reported breast classification. For each volunteer, a radiologist classified the breast as fatty, scattered, heterogeneously dense or dense breast. These classifications correspond to the breast density (% of fibroglandular tissues) of less than 25%, between 25% and 50%, between 50% to 75% and more than 75%, respectively. A high degree of agreement is achieved between calculated breast densities and reported classifications for all the reconstructed models, which can be observed from Table 1. However, a slight discrepancy is found for the Scan A, left breast, where the computed density of the breast classified as scattered is 23.6% (density of Scan A, right breast is 25.4%). Such slight variations are expected as the mammogram based breast classification involves reading of a 2D projected image whereas the breast density computations are based on 3D segmented breast regions.

## Usage Notes

The model files can be easily imported in any numerical scientific computing software or computational electromagnetic software as solid or voxelized models. When importing a solid model, look for the ‘Import Model’ (or similar option) and select STL model format. The voxelized model can also be imported in a similar manner by selecting ‘Import Voxels’ (or similar option). Once the models are imported, electromagnetic properties of the clusters can be assigned in simulation settings. Select single model element (cluster) and choose ‘Single Pole Debye Model’ option in the material settings. Enter the values of the Debye parameters included in the property files corresponding to that cluster. In a similar way, assign properties to all model elements.

Alternatively, the model import process and the assignment of dielectric properties to the model elements can be automated through scripts. A MATLAB based sample script named ‘scriptGenerateModelInfo.m’ (ExampleScripts, Data Citation 1) along with its associated functions are included to help users learn about the usage of the repository files. The script requires the user to select the model, model side, and number of clusters. The users can also choose between the property levels from low, medium or high depending upon the desired dielectric contrast between the breast tissues. It then constructs the electromagnetic breast model by automatically importing all model elements and assigning them the corresponding dielectric properties. This script was also used to generate all the information and figures included in the Model_Info.pdf (Data Citation 1) document available with each breast model.

Another script named ‘ImportModel_Binary.py’ (ExampleScripts, Data Citation 1) is written in python, shows an example of building a breast phantom from binary model files (BinaryModel) in a commercial full wave electromagnetic software. In this case the software used is SEMCAD, however, similar scripts can support model import in other softwares. Similarly, another script named ‘ImportModel_HDF5.py’ (ExampleScripts, Data Citation 1) is provided as an example to import HDF5 format model files using python. A summary of the available sample scripts, dependency functions, script functionality and languages are included in Table 3.

## Additional information

**How to cite this article**: Omer, M. *et al*. Anthropomorphic breast model repository for research and development of microwave breast imaging technologies. *Sci. Data*. 5:180257 doi: 10.1038/sdata.2018.257 (2018).

**Publisher’s note**: Springer Nature remains neutral with regard to jurisdictional claims in published maps and institutional affiliations.

## Supplementary Material



## Figures and Tables

**Table 1:** Study input, processing performed and output data.

Study Input	Processing						Data Output	
**MRI Scan ID**	**Scanner**	**Field Strength**	**Slice dimension×thickness (mm**^**3**^)	**Radiologist Reported Breast Classification**	**Breast Health**	**Constructed Models**	**Voxel size (mm**^**3**^)	**Model density (%)**
Scan A	Siemens Sonata	1.5 T	0.4297 × 0.4297 × 1.2	Fatty – less than 25%	Left - Grade II/III Metaplastic Carcinoma Right - Healthy	BM_Fatty_001	0.43	~13%
Scan B	GE Discovery MR750	3.0 T	0.9375 × 0.9375 × 1.1	Scattered – 25%–50%	Healthy	BM_Scattered_001	0.47	~25%
Scan C	GE Discovery MR750	3.0 T	0.9375 × 0.9375 × 1.1	Scattered – 25%–50%	Healthy	BM_Scattered_002	0.47	~47%
Scan D	GE Discovery MR750	3.0 T	0.9375 × 0.9375 × 1.1	Heterogenous – 50%–75%	Healthy	BM_Hetero_001	0.47	~56%
Scan E	GE Discovery MR750	3.0 T	0.9375 × 0.9375 × 1.1	Dense – more than 75%	Healthy	BM_Dense_001	0.47	~87%
Scan F	GE Discovery MR750	3.0 T	0.9375 × 0.9375 × 1.1	Fatty – less than 25%	Healthy	BM_Fatty_002	0.47	~1.5%
The MRI scans were acquired at Foothills Hospital of the University of Calgary, using a fat-suppressed scan sequence and were processed using 3D breast modelling method ^14^ to construct numerical breast models. Each model is available in multiple file formats, including STL, MAT, RAW and HDF5.								

**Table 2:** Available model formats, their corresponding types and compatibility.

Model Parameter	Model Format	Model Type	Compatibility
Model data	MAT	Voxelized	Matlab
	STL	Stereolithographic	Most commercial EM simulation software
	RAW	Binary	Most custom and commercial EM simulation software
	HDF5	Voxelized	Most custom and commercial EM simulation software
Model properties	XML	Applies to all model types	Most custom and commercial EM simulation software

**Table 3:** Example scripts to demonstrate model import, construction and visualization in different languages.

Script Type	Name	Language	Functionality
**Main**	generateModelInfo	Matlab	Constructs a complete EM numerical breast model from voxelized model elements. It also generates all the information and plots that are included in the Model_Info.pdf file.
	importModel_Binary	Python	Constructs a complete EM numerical breast model from binary model elements.
	importModel_HDF5	Python	Code snippet to show how to read breast model in HDF5 file format.
**Function**	parse_XML_PropFile	Matlab	Imports model properties from XML based property files.
	debyeParamsToEpsSigma	Matlab	Converts Debye model parameters to dielectric property values at selected frequencies.
	getDebyeParams	Matlab	Get tissue specific dielectric properties as Debye parameters.
	volumeVisualization3D	Matlab	Generated 3D visualization of model elements.
The examples include both main script and its dependencies.			
